# Patient-Derived Organotypic Epithelial Rafts Model Phenotypes in Juvenile-Onset Recurrent Respiratory Papillomatosis

**DOI:** 10.3390/v13010068

**Published:** 2021-01-06

**Authors:** Mary C. Bedard, Marion G. Brusadelli, Adrean Carlile, Sonya Ruiz-Torres, Hannah Lodin, Denis Lee, Matthew Kofron, Paul F. Lambert, Adam Lane, Najim Ameziane, El Mustapha Bahassi, Kathryn A. Wikenheiser-Brokamp, Alessandro de Alarcon, David F. Smith, Susanne I. Wells

**Affiliations:** 1 Division of Oncology, Cincinnati Children’s Hospital Medical Center, Cincinnati, OH 45229, USA; mary.bedard@cchmc.org (M.C.B.); Adrean.Carlile@cchmc.org (A.C.); Sonya.Ruiz-Torres@cchmc.org (S.R.-T.); 2 Medpace, Cincinnati, OH 45227, USA; brusadelli.marion@gmail.com (M.G.B.); E.Bahassi@medpace.com (E.M.B.); 3 Mayo Clinic Alix School of Medicine, Mayo Clinic, Phoenix, AZ 85259, USA; HannahLodin@gmail.com; 4 McArdle Laboratory for Cancer Research, University of Wisconsin School of Medicine and Public Health, Madison, WI 53705, USA; Denis.Lee@wisc.edu (D.L.); Lambert@oncology.wisc.edu (P.F.L.); 5 Division of Developmental Biology, Cincinnati Children’s Hospital Research Foundation, Cincinnati, OH 45229, USA; Matthew.Kofron@cchmc.org; 6 Department of Pediatrics, University of Cincinnati College of Medicine, Cincinnati, OH 45267, USA; Adam.Lane@cchmc.org; 7 Division of Bone Marrow Transplantation and Immune Deficiency, Cincinnati Children’s Hospital Medical Center, Cincinnati, OH 45229, USA; 8 Centogene, Am Strande 7, 18055 Rostock, Germany; Najim.Ameziane@centogene.com; 9 Division of Pathology & Laboratory Medicine and The Perinatal Institute Division of Pulmonary Biology, Cincinnati Children’s Hospital Medical Center, Cincinnati, OH 45229, USA; Kathryn.Wikenheiser-Brokamp@cchmc.org; 10 Department of Pathology & Laboratory Medicine, University of Cincinnati College of Medicine, Cincinnati, OH 45267, USA; 11 Division of Pediatric Otolaryngology–Head and Neck Surgery, Cincinnati Children’s Hospital Medical Center, Cincinnati, OH 45229, USA; 12 Department of Otolaryngology–Head and Neck Surgery, University of Cincinnati College of Medicine, Cincinnati, OH 45267, USA; 13 Division of Pulmonary and Sleep Medicine, Cincinnati Children’s Hospital Medical Center, 3333 Burnet Ave. MLC 2018, Cincinnati, OH 45229, USA

**Keywords:** JoRRP, 3D organotypic rafts, low-risk HPV

## Abstract

Juvenile-onset recurrent respiratory papillomatosis (JoRRP) is driven by human papillomavirus (HPV) low-risk strains and is associated with significant morbidity. While previous studies of 2D cultures have shed light on disease pathogenesis and demonstrated the utility of personalized medicine approaches, monolayer cultures lack the 3D tissue architecture and physiology of stratified, sequentially differentiated mucosal epithelium important in RRP disease pathogenesis. Herein we describe the establishment of JoRRP-derived primary cell populations that retain HPV genomes and viral gene expression in culture. These were directly compared to cells from matched adjacent non-diseased tissue, given the known RRP patient-to-patient variability. JoRRP papilloma versus control cells displayed decreased growth at subconfluency, with a switch to increased growth after reaching confluency, suggesting relative resistance to cell-cell contact and/or differentiation. The same papilloma cells grown as 3D organotypic rafts harbored hyperproliferation as compared to controls, with increased numbers of proliferating basal cells and inappropriately replicating suprabasal cells, mimicking phenotypes in the patient biopsies from which they were derived. These complementary model systems provide novel opportunities to elucidate disease mechanisms at distinct stages in JoRRP progression and to identify diagnostic, prognostic and therapeutic factors to personalize patient management and treatment.

## 1. Introduction

Recurrent respiratory papillomatosis (RRP) is a hyperproliferative condition of regenerative epithelial papillomas that occur along the respiratory tract, often requiring repeated surgical removal [1]. The persistent nature of the disease leads to a particularly high disease burden and sometimes fatal complications [2]. Clinical presentation of RRP is divided into two distinct subgroups, adult-onset (AoRRP) and juvenile-onset (JoRRP) disease [3]. JoRRP is etiologically associated with low-risk strains of human papillomavirus (HPV) acquired by vertical transmission at birth [4,5]; nearly all patients with JoRRP are positive for HPV6 or HPV11, with greater prevalence for HPV6 [6]. Conversely, AoRRP has a higher frequency of HPV-negative papillomas [6] and may involve extended viral latency [7]. Additionally, JoRRP is more common and more aggressive than AoRRP [8], with JoRRP patients undergoing 4–5 surgeries on average in the year following diagnosis [9]. As such, JoRPP is the most common benign neoplasm of the larynx in children [3].

The ability to study authentic RRP characteristics, to identify mechanisms driving disease maintenance and progression, and to design effective treatment modalities was previously limited by the lack of reproducible cell culture models. To fill this gap, conditionally reprogrammed cell culture systems were established using primary cells from patients with RRP to support descriptive and mechanistic studies of disease [10,11]. Conditional reprogramming using ROCK inhibitor allows the rapid establishment of patient-derived primary cell cultures with cells maintained in a stem-like state for extended passaging while retaining the ability to differentiate [10,11]. Previous studies have used these cultures of primary papilloma cells to identify differentially expressed disease targets and screen candidate therapeutics [12,13,14]. Individualized cases of successful translation to clinical care were reported, serving as proof of concept for personalized medicine approaches to RRP treatment [12,13]. Unfortunately, no single agent was effective at eradicating RRP, and patient responses to adjuvant therapy remain highly variable and unpredictable [15]. Together, the need for multiple non-curative surgeries and the lack of effective therapeutic options places a burden on patients’ quality of life and the healthcare system.

Despite the association of HPV with disease pathogenesis, infection with HPV does not necessarily result in RRP, and it is unclear which viral components and/or cellular factors elicit the clinical phenotype [2]. In patient biopsies, HPV-related pathological changes are associated with the expansion of the basal layer with ectopically proliferating cells in the suprabasal compartment and with viral associated cellular alterations (referred to as koilocytic changes) in the superficial layers of the stratified epithelium. Interestingly, only scattered HPV positive epithelial cells are identified in papilloma biopsies by routine clinical assays, raising questions as to which morphologic and cellular HPV-related phenotypes are a direct result of viral infection and replication. Additionally, it is unclear if the variability in patient presentation is related to specific viral factors and if disease progression requires continued HPV infection and replication. An ideal model system would enable elucidation of HPV-driven cellular disease mechanisms as well as testing of drug efficacy in individual patient-derived matched RRP and non-diseased differentiating stratified epithelium to maximize therapeutic benefit while minimizing toxicity. To this end, RRP xenograft models were developed for studies of drug sensitivity [16] with variable success [17]. In studies of high-risk HPV, 3D organotypic rafts are frequently used to recapitulate in vivo viral features and to enable mechanistic studies and drug screening [18,19,20]. However, similar model systems to comprehensively study low-risk HPV induced pathogenicity in RRP-derived cells have not been reported to date.

Herein, we demonstrate the importance of utilizing 3D models in addition to traditional 2D cell culture to study the biological features of JoRRP. We report that primary cultures of papilloma and matched non-diseased primary keratinocytes can be consistently established from small biopsies obtained during routine standard-of-care excisions. Papilloma-derived primary cells retain HPV genomes as well as viral gene expression, providing a system to identify viral dependent cellular disease mechanisms. Moreover, we demonstrate that these primary cell cultures form corresponding 3D organotypic rafts that mimic the regional specific hyperproliferative phenotypes present in the patient biopsies from which the cells were derived. Taken together, our studies demonstrate that 2D cultures of primary papilloma versus non-diseased cells are optimally complemented by 3D organotypic rafts to provide a pipeline for expanding knowledge of JoRRP pathogenesis and identifying personalized therapeutics.

## 2. Materials and Methods

### 2.1. Study Participants and Biopsy of RRP Lesions

Patients 1 to 17 years of age with a history of JoRRP were previously recruited to a prospective study through the Cincinnati Children’s Hospital Medical Center Division of Pediatric Otolaryngology—Head and Neck Surgery. Approval was obtained from the Institutional Review Board (IRB study ID 2015-1303). Each patient underwent microlaryngoscopy and bronchoscopy after recruitment as part of the standard of practice to evaluate for papillomas in the airway. Prior to surgical excision, microsurgical instruments were used to obtain biopsies of the laryngeal papillomas and adjacent normal control tissue. Specimens were specifically taken from papillomas of the larynx, isolated to the supraglottis, glottis, or subglottis. A biopsy from the same patient was also taken from normal tissue along the posterolateral arytenoid mucosa where no gross disease was present to obtain papilloma-normal matched biopsy sets for all patients. The biopsy specimens were placed in DMEM/F12 (11-320-033 Thermo Fisher Scientific, Waltham, MA, USA) on ice and immediately processed. Tissues were bisected and divided for formalin-fixed paraffin-embedded (FFPE) sections and for the establishment of primary cell cultures. In cases where insufficient tissue was available at the time of recruitment surgery, the establishment of a tissue bank of FFPE specimens for morphological analysis was prioritized, with cultures established from tissue obtained at subsequent surgeries for most study participants. The Derkay score, a validated clinical staging system [21,22], was determined from recorded laryngoscopy videos. The Derkay score includes both a subjective assessment of clinical parameters as well as an anatomic assessment of disease distribution by subsite of the larynx, trachea and respiratory tract.

### 2.2. Mucosal Squamous Epithelial Cell Culture

Specimens were treated twice with fresh 1× PBS containing 10% Pen/Strep and 5% Fungizone for 3 min, washed in 1× PBS, and transferred to a 60 mm (mm) culture dish containing 0.25% trypsin/1× EDTA. Samples were dissected into small fragments using surgical tools, resuspended vigorously by rapid pipetting, and incubated for 10–15 min at 37 °C. Trypsin was inactivated with 2.5× volumes F-media, and the samples were centrifuged at 1200 rpm for 3 min. Media was aspirated, and samples were resuspended in complete F-media supplemented with 10uM of Y-27632 ROCK inhibitor (ALX-270-333-M025 Enzo Life Sciences, Farmingdale, NY, USA) and plated onto irradiated feeder fibroblasts as previously described [10,11]. Monolayer cell culture was considered successful if robust keratinocyte outgrowth of primary cells, considered passage 0 (p0), was observed within 10 days after plating. Cells were passaged upon reaching 70% confluency and successfully cultured for at least 10 passages. Cell pellets were collected from early passage (p3–p6) primary cells.

### 2.3. Organotypic Epithelial Raft Culture

Organotypic epithelial rafts were generated as previously described as papilloma/non-diseased matched primary cells for each patient [18]. Briefly, 2 × 10^6^ mucosal epithelial cells were plated on a collagen matrix that harbors embedded fibroblasts. 10 μM of Y-27632 ROCK inhibitor was added to keratinocyte plating media for the first four days of culture. After four days of submerged growth, introduction to an air-liquid interface and high calcium media promoted the generation of stratified epithelium with differentiation properties that reflect human squamous epithelium. 10 μM 1,2-dioctanoyl-sn-glycerol (C8:0) (62225 Cayman Chemical Company, Ann Harbor, MI, USA) was added to cornification media to induce more complete differentiation and HPV gene expression [23,24]. Rafts for all reported experiments were harvested 10 days after air-lifting (day 14 total). Rafts used for histology were embedded in paraffin. A slide from each set of sections was stained with hematoxylin and eosin and examined for histopathology by a board-certified pathologist.

### 2.4. Quantitative Reverse Transcription PCR (RT–qPCR)

RNA was isolated from sets of papilloma/non-diseased 2D cell pellets using the RNeasy Mini Kit (74106 Qiagen, Hilden, Germany). For each sample, the quality of extracted RNA was confirmed by NanoDrop to have a 260/280 ratio of 2.0 or above, and 1 µg RNA was reverse transcribed to cDNA using the QuantiTect reverse transcription kit (205314 Qiagen) according to the manufacturer’s instructions. PCR primers were developed based on PaVE sequences [25] (http://pave.niaid.nih.gov) using Primer3web version 4.1.0 and Primer-BLAST [26] and purchased from IDT. The PCR reaction mixture (20 µL) contained 10 ng cDNA, 10 µL SYBR green master mix (A25742 Thermo Fisher), 1 µL each of forward and reverse primer, and 3 µL of RNAse-free water. RT–PCR experiments were run on an ABI7500 Applied Biosystems Step One Plus real-time PCR system with StepOne software v2.2 (Applied Biosystems, Foster City, CA, USA). Assays were performed in accordance with the manufacturer’s instructions. Primers did not have a signal in non-targeted control wells and had one peak in the melting curve. CT values above 35 were excluded. Data were analyzed by calculating the–ΔCt with β-actin as the housekeeping gene (where ΔCt represented the Ct of viral gene minus Ct of housekeeping). Higher values on the corresponding graphs, therefore, corresponded to a higher magnitude of gene expression. Sequences of primers used were as follows: β-actin (forward: 5′-AGA GCT ACG AGC TGC CTG AC-3′and reverse: 5′-AGC ACT GTG TTG GCG TAC AG-3′), HPV6 E1 (forward: 5′-CAC TAT AGC CGA GGC AGT GG-3′and reverse: 5′-GCA GCC CTG TAT TGG TTT CG-3′), HPV6 E6 (forward: 5′-TGG AAA GTG CAA ATG CCT CC-3′and reverse: 5′-CTC TGC TGT GGT CAG TGC A-3′), HPV6 E7 (forward: 5′-GGA CGA AGT GGA CGG ACA AG-3′and reverse: 5′-CAC TGC ACA ACC AGT CGA AC-3′), HPV6 L1 (forward: 5′-GGT TAT CGC CTC CCC CAA AT-3′and reverse: 5′-GGA GTG GGC TTT TGA CAG GT-3′), and HPV6 L2 (forward: 5′-GTT GGG TAT AGG CAC GGG TT-3′ and reverse: 5′-GCC CCT GCG TTA ATG ATT GC-3′).

### 2.5. Determination of HPV Strain by RT–qPCR

RT–qPCR was performed as above with primer sets specific to HPV6 E6 and HPV11 E6. Primer sets were designed as described above and confirmed for HPV strain specificity using the PaVE’s PV-specific BLAST [25] and purchased from IDT. Sequences of primers for HPV6 E6 are listed above; HPV11 E6 sequences were as follows: forward: 5′ ACC TGT GTC ACA AGC CGT TG and reverse: 5′ AGC AGT GTA AGC AAC GAC CC.

### 2.6. Southern Blot Analysis

Total genomic DNA was extracted from matched 2D papilloma/non-diseased samples using Qiagen’s DNeasy Blood and Tissue kit. A total of 5 µg DNA was digested with Bam HI, an enzyme that will linearize the HPV 6 genome. These digested DNAs were electrophoresed on a 0.8% agarose gel along with an HPV 6-containing plasmid that was digested with Bam HI to release the viral genome as a standard. After electrophoresis, these DNAs were transferred to the Hybond N^+^ membrane (GE Healthcare, Amersham, Buckinghamshire). The membrane was then probed with a set of 20 oligos complementary and specific to HPV 6 that was labeled with γ-^32^P-ATP. To visualize HPV 6 DNA, the washed membrane was exposed to a PhosphorImager screen that was then scanned using a Typhoon (GE Healthcare).

### 2.7. 2D Culture on Coverslips with EdU Incorporation

Glass coverslips (Thermo Fisher 12-545-80) were coated with poly-L-lysine (P0899 Sigma-Aldrich, St. Louis, MO, USA) and dried according to manufacturer directions prior to placing in 6-well tissue culture plates in media (11-965-118 Thermo Fisher). Irradiated feeder cells were plated on coverslips one day prior to plating 100,000 primary cells per well. Cells were plated in sets of papilloma/non-diseased primary cells in parallel per condition (subconfluency and overconfluency) and monitored daily. Cells were harvested at 40–50% confluency for the subconfluent condition. Once cells reached confluency at 4–5 days, the media was changed and subsequently replaced every 24 h. 10 μM EdU was added to media 2 h before harvest, whereupon coverslips were transferred to a fresh 6-well plate, fixed for 30 min in 4% paraformaldehyde, washed three times with 1× PBS, and stored in 1× PBS until use.

### 2.8. Immunofluorescence Staining

For organotypic raft and tissue sections, slides were deparaffinized, and antigen retrieval was performed in 10 mM sodium citrate pH 7 for 15 min at 110 °C in a decloacker chamber (Biocare, Pacheco, CA, USA). For 2D coverslips, permeabilization was performed with 0.2% Triton X-100 for 3 min at room temperature (RT). Subsequent steps were performed in a humidity chamber with the slides protected from light. To stain for EdU incorporation, the Click-it EdU cell proliferation assay kit (C10640 Invitrogen, Carlsbad, CA, USA) was used per the manufacturer’s instructions. Samples were blocked with 10% donkey serum (017-000-121 Jackson ImmunoResearch Laboratories, West Grove, PA, USA) in PBS for 1 h at RT. Primary antibodies (collagen 17, abcam ab184996, 1:200; cornulin, R&D systems AF3607, 1:200; k(eratin) 14, BioLegend 905301, 1:2000; E-cadherin, R&D systems AF648, 1:200; Ki67, Abcam ab238020, 1:200) were diluted in antibody dilution buffer (3% BSA, 0.5% Triton-X100, 0.05% Tween-20, 0.04% sodium azide in PBS) and 2% donkey serum and incubated on slides for 1 h at 37 °C. Secondary antibodies (Jackson ImmunoResearch Laboratories) were diluted in antibody dilution buffer with 0.5 µL 4′,6-diamidino-2-phenylindole (DAPI) (D1306 Thermo Fisher) and incubated on slides for 30 min at 37 °C. For membrane staining of organotypic rafts, wheat germ agglutinin (W849 Thermo Fisher) was added to the secondary antibody solution. Prolong gold (P36934 Thermo Fisher) was used for mounting. Slides were stored at 4 °C until imaging and at 20 °C for long-term storage.

### 2.9. Confocal Imaging

All confocal images were taken on a Nikon A1 confocal scan head with GaAsP detectors on a TiE inverted microscope. Patient biopsies were imaged using a 10× Plan Apo objective; all other samples were imaged using a 20× Plan Apo objective and a 1.2AU pinhole giving a 3.1 um optical section. Z-stacks were acquired by determining the upper and lower boundaries of the samples and scanning optical sections with the overlap of adjacent sections. Z-stack ranges were approximately 10 µM for subconfluent cells or 3D organotypic rafts and 20 µM for overconfluent cells. Laser and PMT voltage (gain) settings were set for each type of sample and stain separately, then kept consistent between matched papilloma/non-diseased specimens. For 2D coverslips with subconfluent cells, every epithelial colony visible by DAPI on one coverslip per papilloma/non-diseased sets was imaged. For 2D coverslips with overconfluent cells, a standardized list of xy locations was predefined across the area of a coverslip and set relative to each coverslip imaged. Frames were confirmed to have cells by visualization of DAPI by eye, and xy locations removed per coverslip that showed damage or refraction due to proximity to the coverslip edge. For 3D organotypic rafts and patient biopsies, the entire area of the specimen was imaged. Maximum intensity projections (MaxIP) of Z-stacks were created in NIS elements followed by artificial intelligence denoising (denoiseAI) to eliminate shot noise. Processed MaxIP.nd2 files exported as composite and single-channel TIF files were used for visualization and quantification.

### 2.10. Quantification of EdU and DAPI-Positive Cells

EdU+ cells per frame in 3D organotypic rafts were counted manually, with the exclusion of any fibroblasts embedded in the underlying collagen matrix. For 2D coverslips, single-channel EdU and DAPI TIF images were processed in ImageJ [27] using the standardized macro [run(“16-bit”); setAutoThreshold(“Default dark”); //run(“Threshold...”); run(“Analyze Particles...”, “size=30-Infinity show=[Overlay Masks] summarize”); with output set to jpeg files]. Images containing a particle size of over 500 pixels were reviewed separately. Frames close to the edge of coverslips had light dispersion with low contrast/high background that caused inaccuracies in automated ImageJ counting, as did particle sizes above 500, and thus both DAPI and EdU counts for these frames were removed from the analysis. Overlay counts in output jpeg files were reviewed to ensure accurate counting, and the counts of randomly selected images were confirmed manually. A total of 44–59 frames per subconfluent coverslip and 38–51 frames per overconfluent coverslip were verified for analysis.

### 2.11. Statistical Methods

**Figure 1:**
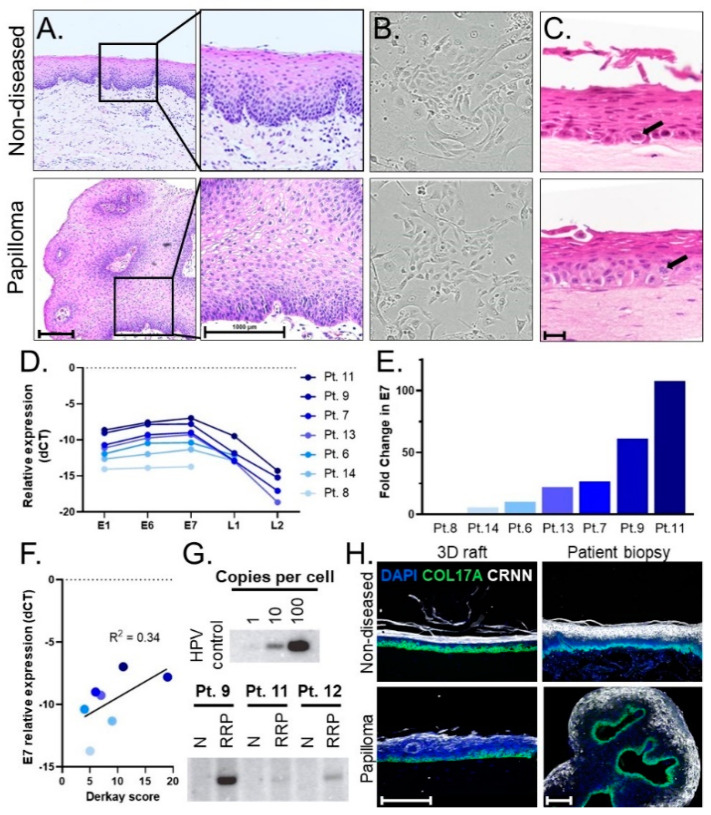
(**A**) Histology of pediatric laryngeal papilloma and non-diseased tissue biopsy (H&E stain). Scale bar, 1000 µm. (**B**) 10× images of papilloma and non-diseased primary keratinocyte cells cultured from matched patient biopsies exhibiting healthy growth and normal morphology. (**C**) Representative images of 3D organotypic rafts generated from matched primary cells. An ectopic mitotic figure (arrow) is shown in a raft generated from papilloma derived cells as compared to the normal location of the mitotic figure (arrow) in the basal layer of the raft generated from non-diseased tissue-derived cells. Scale bar, 20 µm. (**D**) Relative expression (-dCT values normalized to β-actin) of HPV viral genes using matched primary cells from 7 patients, with L1 and/or L2 levels in 3 patients below the limit of detection. Higher values (smaller negative numbers) corresponded to higher viral gene expression. (**E**) Fold change of E7 gene expression is expressed relative to patient 8, who harbors the lowest E7 expression levels. (**F**) Scatterplot showing positive correlation (r-squared 0.34) between the level of E7 gene expression and Derkay scores as a measure of disease severity. (**G**) Southern blots for HPV6 genome detection showing genome copies in papilloma (RRP), but not non-diseased (N), primary cells with high patient variability in viral load. (**H**) Epithelial layers present in the stratified epithelium of patient biopsies are modeled in derivative 3D organotypic rafts. Basal layer, COL17A (green); differentiated layer, CRNN (white); cell nuclei, DAPI (blue). Scale bar, 100 µm.

All statistics were performed using GraphPad Prism6 software. For data within one patient, data are represented as the mean ± standard deviation. For data across several patients, data are represented as the mean ± standard error of the mean. To determine statistical significance, Student’s t-test with two-tailed distribution was used when comparing two ratio variables (Figure 3E). Otherwise, two-way ANOVA with multiple comparisons was used. Statistical significance was set as follows: * = *p* < 0.05, ** = *p* < 0.01, *** = *p* < 0.001. Linear regression was performed when comparing ratio variables (Figure 1F), with goodness-of-fit evaluated by R-squared. For each experiment, the number of replicates and the *p* values are noted in figure legends.

## 3. Results

To perform comprehensive studies of disease pathogenesis, we recruited 26 children with JoRRP and obtained papilloma/non-diseased matched biopsies during routine surgical interventions. In addition to 2D primary cell culture, we hypothesized that 3D models routinely used in studies of high-risk HPVs [11] might broaden the range of tools available for mechanistic studies, therapeutic screening, and personalized medicine approaches for JoRRP.

### 3.1. Establishing a Pipeline of Patient-Derived 2D Primary Cells and 3D Organotypic Rafts to Study JoRRP

We established a pipeline of patient biopsies, patient-derived 2D primary cells, and 3D organotypic rafts as models to study non-diseased and papilloma stratified squamous epithelium. Following surgical excision, matched papilloma/non-diseased biopsies were processed for review by a clinical pathologist. Compared to non-diseased tissue, papilloma specimens displayed thickened stratified squamous epithelium and HPV viral cytopathic effects, collectively known as koilocytic atypia, including nuclear enlargement, hyperchromasia, abnormal variations in size and shape, and cytoplasmic perinuclear halos that reflect cytoplasmic vacuolization (Figure 1A). Additional matched papilloma/non-diseased biopsies were used to establish primary monolayer cultures. Successful cultures were established from 19 of 26 patients, with insufficient amounts of tissue available from the remaining patients for culture. The resulting 2D populations exhibited robust cell growth and were passaged at least ten times without exhaustion (Figure 1B) by using a ROCK inhibitor that was shown previously to extend the lifespan of many epithelial cell types in culture [10,11]. Matched papilloma/non-diseased cells from three patients were then used to generate 3D organotypic rafts [18] that recapitulated the structure of the human epithelium (Figure 1C). Both papilloma and non-diseased primary cells generated stratified squamous epithelial rafts with architecture and progressive directional squamous differentiation, recapitulating the histology in the corresponding patient biopsies from which they were derived. Rare mitotic cells were identified in both papilloma and non-diseased control rafts. Notably, the rare mitotic figures were limited to the basal layer in the non-diseased control rafts but were also identified in the suprabasal layers in the papilloma rafts, suggesting that ectopic proliferation contributed to the papilloma phenotype.

Early passage cell cultures were tested for their ability to retain HPV. First, HPV6 versus HPV11 infection was determined for each patient. Primary cell pellets were analyzed by RT-qPCR using HPV6- and HPV11-specific primers. Papilloma/non-diseased primary cells from nineteen patients were analyzed to date, with seventeen HPV6+ and two HPV11+. No papilloma cells tested positive for both HPV6 and 11, and no signal was detected in non-diseased primary cells, verifying the absence of viral infection in patient-matched control samples. Primary cells from seven HPV6-positive patients were evaluated by RT-qPCR for HPV gene expression of E1, E6, E7, L1, and L2. HPV viral gene expression was only observed in papilloma cell populations. The relative expression of HPV genes (E1≈E6≈E7 > L1 > L2) was notably consistent across patients (Figure 1D). Overall, 2D primary cells expressed high levels of early HPV genes and lower levels of late genes L1/L2. This finding is consistent with the function of early versus late HPV viral genes; early genes are associated with viral maintenance, replication, and disease pathogenesis while late genes are involved in virion production and packaging [4]. To highlight the range in viral gene expression across patients, fold change relative to the patient with the lowest expression (patient 8) was calculated for E7 (Figure 1E). The patient with the highest E7 expression (patient 11) had a 107-fold increase in E7 as compared to patient 8. Relative gene expression was evaluated in the context of disease severity of patients in the cohort. To this end, we compared gene expression to patients’ Derkay scores, a clinical measure that assesses disease burden by subsite in the aerodigestive tract. While a positive correlation was found between HPV gene expression and disease severity as measured by the Derkay score, the R-squared of 0.34 indicated that other variables also contribute to disease severity (Figure 1F). HPV viral DNA genomes were detected in papilloma—but not in non-diseased—cells by Southern blot analysis and showed significant variation in genome copy number among patients that was surprisingly not correlated with viral gene expression (Figure 1G). We conclude that papilloma-derived primary cells maintain HPV DNA genomes and viral RNA expression with high variability of genome load and viral gene expression, which may reflect the variable clinical course and treatment history among patients with JoRRP.

We then evaluated the tissue architecture of 3D organotypic rafts derived from papilloma/non-diseased patient cells using biomarkers for stratified epithelium (Figure 1H). The expression of collagen 17 (COL17A) and cornulin (CRNN) are known to be specific for the basal layer and differentiated layers, respectively [28,29]. Accordingly, staining for these markers confirmed the organization of 3D organotypic rafts into the stratified squamous epithelium. Importantly, the 3D organotypic rafts formed stratified squamous epithelial mucosa with a basal layer and differentiation in superficial layers comparable to the corresponding tissues of origin. Altogether, we established a patient-derived pipeline of 2D primary cells that retain HPV and form 3D organotypic rafts that recapitulate the mucosal epithelium.

### 3.2. Papilloma versus Non-Diseased Primary Cells Exhibit Diminished Growth in Standard 2D Tissue Culture

**Figure 2:**
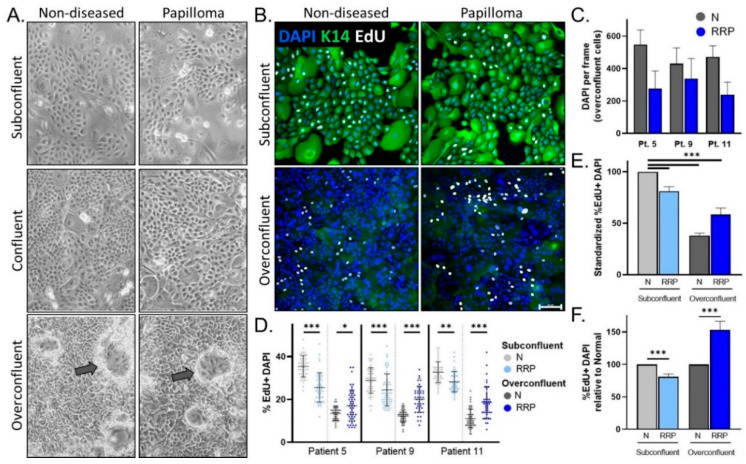
(**A**) Representative 10× images of 2D matched papilloma and non-diseased primary cells at subconfluency (top), confluency (middle), and overconfluency (bottom) states. Arrows depict differentiating cells growing upwards. (**B**) Immunofluorescence (IF) analysis for the basal cell marker K14, EdU, and DAPI in papilloma and matched non-diseased cells. Scale bar 100 µm. (**C**) Quantification of DAPI nuclei per frame at overconfluency demonstrates intra-sample variability and a trend to decreased DAPI counts in matched papilloma (RRP) versus non-diseased (N) primary cells. (**D**) Percentage of EdU+ DAPI+ nuclei in papilloma and non-diseased cells at subconfluency (n = 44–59 frames) and overconfluency (n = 38–51 frames) across three patients. (**E**) Average% EdU+ DAPI+ nuclei across patients relative to non-diseased cells in standard cell culture conditions (subconfluency) show a decrease in proliferation after reaching confluency. (**F**) Compared to non-diseased cells, papilloma cells have slightly decreased EdU incorporation (19%) at subconfluency, whereas their proliferation significantly increases (53%) at overconfluency. * = *p* < 0.05, ** = *p* < 0.01, *** = *p* < 0.001.

We next tested whether hyperproliferation, a hallmark of JoRRP [1], is modeled in primary cells and/or organotypic rafts. During routine passaging of cells starting with equal cell counts, it was empirically observed that papilloma-derived cultures took longer than non-diseased tissue-derived cells to reach confluency. We selected papilloma/non-diseased primary cultures from two HPV6+ patients (patients 9 and 11) and one HPV11+ patient (patient 5). We quantified proliferation in matched papilloma and non-diseased cells from these three patients during the growth phase (subconfluent cells) and two days past confluency (overconfluent cells) (Figure 2A). To quantify proliferating cells, EdU incorporation experiments were performed and visualized by confocal microscopy analysis using K14 to highlight cell morphology (Figure 2B). As expected from Figure 2A, primary cells flattened and started to differentiate after reaching confluency, resulting in a dense culture of squamous epithelial cells with decreased expression of K14. Notably, there were consistently fewer papilloma cells at overconfluency when compared to non-diseased cells (Figure 2C). To account for variation in the number of cells per field, the number of EdU+ cells in a frame was therefore normalized to DAPI nuclei. Consistent results were observed across all patients, with 29–35% of non-diseased cells and 24–28% of papilloma cells showing EdU incorporation at subconfluency, and 11–13% and 17–20%, respectively, at overconfluency (Figure 2D). Papilloma versus non-diseased cells had decreased proliferation at subconfluency (*p* < 0.001), consistent with the observed delayed time to confluency (Figure 2E). While non-diseased cells responded to overconfluency with a dramatic reduction in proliferation in line with differentiation, papilloma cells responded with a lesser reduction. As a result, the relationship observed between papilloma and non-diseased cell proliferation at subconfluency was reversed at overconfluency, with an average of a 19% decrease in subconfluency (*p* < 0.001) and 53% increase in overconfluency proliferation (*p* < 0.001) in papilloma cells (Figure 2F). Overall, these data demonstrate that subconfluent standard 2D papilloma cells do not recapitulate RRP hyperproliferation unless an overconfluent state is reached.

### 3.3. Engineered 3D Organotypic Raft Models Recapitulate Disease Phenotypes Observed in Patient Biopsies

**Figure 3:**
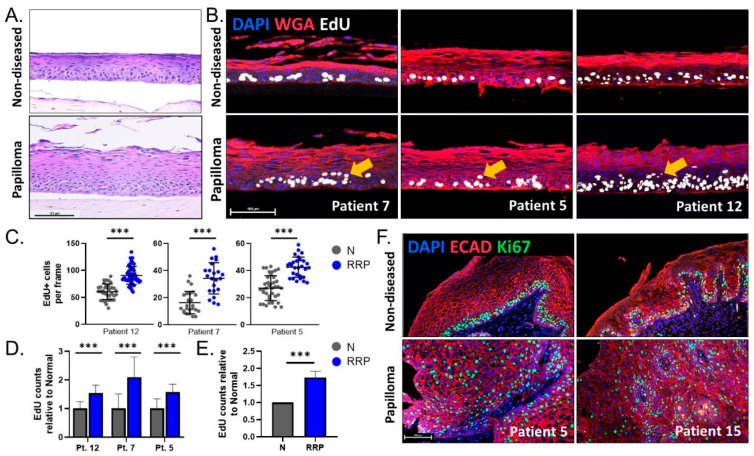
(**A**) Representative H&E of 3D organotypic rafts from patient 12 showing morphological changes in papilloma-derived rafts. (**B**) IF analysis for DAPI (blue), WGA (membrane marker, red), and EdU (white) on 3D rafts from three patients. Arrows indicate suprabasal EdU+ proliferating cells in papilloma-derived organotypic rafts. (**C**) Quantification of EdU+ cells per frame for organotypic rafts depicted in (B) (n = 22–40 frames). Y-axis is adjusted per patient based on the baseline level of proliferation in the non-diseased raft, highlighting intrinsic patient variability. (**D**) EdU counts in papilloma (blue) relative to non-diseased (gray) rafts per patient. (**E**) A relative increase in proliferation of 74% was observed between papilloma and matched non-diseased 3D organotypic rafts. (**F**) IF analysis for DAPI (blue), ECAD (membrane marker, red), and Ki67 (green) for two patients showing increased and abnormal distribution of proliferating cells in papilloma biopsies. Scale bar 100 µm in all figures. *** = *p* < 0.001.

Since overconfluency mimics the formation of 3D tissue via differentiation, we next evaluated hyperproliferation in 3D organotypic rafts derived from papilloma and matched non-diseased primary cells from two HPV6+ patients (patients 7 and 12) and one HPV11+ patient. Patient 12 raft morphology is shown in Figure 3A for reference. Proliferation was measured by EdU incorporation. Papilloma-derived organotypic rafts had significantly more EdU+ proliferating epithelial cells in both basal and suprabasal layers compared to non-diseased controls in all three patients studied (Figure 3B). Total EdU+ cells per frame were then quantified in the papilloma and non-diseased rafts for each patient (Figure 3C). Notably, baseline proliferation in non-diseased organotypic rafts differed significantly between patients, highlighting intrinsic patient-to-patient variability and the importance of comparing papilloma to matched non-diseased controls. Average EdU counts were normalized to matched non-diseased organotypic rafts for each patient and showed a consistent increase in proliferation in papilloma-derived organotypic rafts (54–110%, *p* < 0.001) (Figure 3D). Overall, there was a 74% increase (*p* < 0.001) in proliferation in papilloma-derived organotypic rafts, in line with the hyperproliferative phenotype observed in overconfluent cultures and in the JoRRP tissues (Figure 3E). To validate in vivo tissue phenotypes, papilloma and non-diseased patient biopsies were stained for the proliferation marker Ki67 (Figure 3F). While Ki67+ cells in non-diseased biopsies were confined to the basal layer, papillomas also had ectopically proliferating suprabasal Ki67+ cells. Together, these data demonstrate the capability of 3D organotypic raft models to capture patient variability and recapitulate the hyperproliferative phenotype and ectopic proliferation hallmarks of JoRRP.

## 4. Discussion

Studies using 2D cultures of patient-derived primary cells have made important contributions to our understanding of RRP and disease pathogenesis. Cell culture systems of primary papilloma cells have allowed the identification of candidate therapeutic targets such as Rac1 [12], high-throughput drug screening [14], and the successful application of personalized medicine approaches to treat a lung malignancy resulting from RRP progression [13]. However, a remaining limitation of these and any 2D cell culture system is the inability to model 3D tissue architecture, physiology, and pathology in vitro. Furthermore, the differentiation programming that normally results in regional squamous cell heterogeneity that defines the stratified mucosal epithelium is largely absent in monolayer culture.

To address these limitations, we established matched papilloma/non-diseased 2D cultures from patient biopsies into 2D primary cells and engineered complementary 3D organotypic rafts that modeled a key phenotype in JoRRP, namely increased and ectopic proliferation that defines recurrent papillomas. For the first time, non-diseased tissue from each patient enabled direct comparisons between diseased and non-diseased tissue in an individual patient to address the marked patient variability characteristic of this disease. Interestingly, we found that primary cells derived from papilloma lesions consistently took longer to reach confluency with fewer proliferative cells compared to their non-diseased counterparts. However, as non-diseased cells reached and passed confluency with an expected switch to proliferative arrest during differentiation, papilloma cells exhibited a lesser reduction and persisted in proliferating at overconfluency. Significant proliferative gains in papilloma versus non-diseased cells were also observed in 3D organotypic rafts, with papilloma-derived rafts harboring increased EdU+ basal cells and inappropriate replication in the suprabasal compartment of the epithelium. These findings in 3D organotypic rafts are in line with Ki67 expression in patient biopsies, where proliferating cells are confined to the basal layer of non-diseased biopsies but distributed throughout the epithelium in papilloma biopsies. Taken together, these models reflect JoRRP pathogenesis—a slow and steady persistence of disease-associated cells in driving increased proliferation in differentiating tissue.

Our experiments also revealed two unexpected findings that warrant further study. First, we identified a decreased rate of proliferation in papilloma cells in standard 2D cultures as compared to non-diseased cells. This may be due to an increased sensitivity of papilloma cells to the stress of monolayer culture, compared to their healthy, non-diseased counterparts. However, relevant mechanisms underlying this phenotype remain unclear. Second, we identified significant variation in viral gene expression across patients, and this variation was independent of viral DNA copy number. The degree of viral gene expression showed a positive correlation with the bulk of disease by Derkay score, but the relatively low r-squared of 0.34 indicated the importance of other factors. Additional prospective data will be necessary to establish the clinical progression of patients within the cohort and to explore the effects of HPV gene expression variation on the host tissue due to factors such as exposure to therapies and disease course. The disconnect between viral gene expression and genome load may be due to a limited number of samples or may reveal a deeper, more complicated relationship of the state of HPV replication versus gene expression. Further studies of virus-related factors such as episomal versus integrated states of the HPV genome are needed to explore this relationship between viral gene genome copy number and expression. Additionally, levels of HPV gene transcription and DNA replication vary based on both the productive state of the virus (latent vs. active) as well as the sub-type of squamous cell within the epithelium (basal cells vs. differentiated cells). The lack of a linear relationship between genome copy number and viral gene expression across patients’ primary papilloma cells may reflect the complexity of these factors in addition to patient-specific histories of immunomodulatory and adjuvant treatments.

There are several limitations to this study. First, the cell culture of patient biopsies results in a highly heterogeneous squamous epithelial population, and therefore, viral features in primary derivative cells may not fully represent the original papilloma specimen. While we utilized the earliest passage of cells within logistical considerations, it is possible that epithelial subpopulations specific to the disease, which exhibit slower growth, could be lost with successive passaging. Second, organotypic rafts do not display the same drastic extent of proliferating suprabasal cells present in papilloma biopsies. Due to the relatively short two week time period of organotypic raft generation, we suggest that organotypic rafts represent early stages of papilloma formation rather than fully formed lesions. Other techniques, such as xenograft models, are likely necessary to achieve fully formed papillomas but were reported with variable success by others [16,17] and likely required a more extended culture time.

Taken together, our studies demonstrate that 2D cultures of primary papilloma versus non-diseased cells are ideally complemented by 3D organotypic rafts to mimic morphological and biological features of patient-specific JoRRP lesions. Successful generation of patient-derived HPV infected papilloma and paired non-diseased control squamous epithelial cells and organotypic rafts now provide unique opportunities to study low-risk HPV disease mechanisms and relate them to patient outcomes and response to treatment. Since HPV-related pathologies are often described in the context of the stratified human epithelium, the ability to correlate cellular phenotypes with corresponding 3D models and patient biopsies is a distinguishing positive attribute of this model system. Our established pipeline demonstrates that personalized papilloma and corresponding non-diseased tissue models can be readily generated from small biopsies obtained during routine clinical care. This approach provides an experimentally powerful in vitro model to explore papilloma-specific pathways and relevant mechanisms in the identical genetic environment of patients with JoRRP, with the goal of identifying specific patient-viral interactions. Additionally, the pipeline enables the possibility of personalized medicine approaches that tackle the issue of high clinical variability in disease course and treatment responses. Using this pipeline, potential therapies could be tested in a patient’s own papilloma and non-diseased derivative epithelium to select maximally efficacious drugs with minimal toxicity.

## Data Availability

The published article includes all datasets generated or analyzed during this study.
